# A Digital Twin Approach to a Quantitative Microstructure-Property Study of Carbon Fibers through HRTEM Characterization and Multiscale FEA

**DOI:** 10.3390/ma13194231

**Published:** 2020-09-23

**Authors:** Rebekah Sweat, Jin Gyu Park, Richard Liang

**Affiliations:** Department of Industrial and Manufacturing Engineering, FAMU-FSU College of Engineering High-Performance Materials Institute, Florida State University, Tallahassee, FL 32310, USA; parkji@eng.famu.fsu.edu (J.G.P.); liang@eng.famu.fsu.edu (R.L.)

**Keywords:** multiscale, microscopy, digital twin, carbon fiber

## Abstract

Microstructures of typical carbon fibers (CFs) from polyacrylonitrile (PAN) and pitch-based precursors were studied using a novel digital twin approach with individual carbon fibers for a local crystal scale model. The transmission electron microscopy (TEM) samples were prepared using a focused-ion beam (FIB) for both longitudinal and transverse directions of carbon fibers. Measurements of the crystal size and orientation were estimated from X-ray scattering. TEM imaging of graphitic packing facilitated further comprehension of associations between processing and final material properties, which could enable customization of microstructures for property targets. Then the detailed microstructural information and their X-ray scattering properties were incorporated into the simulation model of an individual carbon fiber. Assuming that graphene properties are the same among different forms of carbon fiber, a reasonable physics-based explanation for such a drastic decrease in strength is the dislocations between the graphitic units. The model reveals critical defects and uncertainty of carbon fiber microstructures, including skin/core alignment differences and propagating fracture before ultimate failure. The models are the first to quantify microstructures at the crystal scale with micromechanics and to estimate tensile and compressive mechanical properties of carbon fiber materials, as well as potentially develop new fundamental understandings for tailoring carbon fiber and composites properties.

## 1. Introduction

Carbon fibers (CFs) are widely used as the reinforcement material in high-performance composite applications because of their lightweight nature and high mechanical properties [1]. Carbon fiber properties are driven by the microstructure generated through precursors and processing [2]. Different precursor materials are used for carbon fiber production. Polyacrylonitrile (PAN) and pitch are the most common precursors for manufacturing [3,4]. After drawing, the precursor materials, oxidization, stabilization, and carbonization processes are performed for carbon fiber production. Depending on the precursor materials and fiber production conditions, a broad range of mechanical strength and modulus can be obtained. Therefore, various CFs can be selected for reinforcement depending on the strength or modulus application needs in a composite fabrication. Much work has shown that as crystallite size reduces, tensile strength increases, and as orientation improves, tensile modulus rises. Defect generation and size are also related to crystal size, which can reduce the tensile strength of carbon fibers with an increase in tensile modulus. Although this correlation is understood for tensile behavior, the microstructural effect on compression and shear behavior of fibers remains mostly empirical [5].

The lack of a fundamental understanding of microstructure property relationships is a significant scientific barrier to the design of a new generation of carbon fibers with tailored and high-performance properties, especially for compressive, tensile, and shear properties. Notably, no digital models have been developed to explore multiple mechanical properties at different loading conditions at the crystal scale level. A digital twin model of carbon fiber with true graphite crystal structures has been embedded in a global single carbon fiber model. A model captured internal microstructures of carbon fibers from atomic to nanoscale crystal and microscale based on advanced transmission electron microscopy (TEM), FIB, and X-ray characterizations. These models developed a new fundamental understanding for tailoring carbon fiber and composites properties. The digital twin approach gives detail and output that experiments cannot. X-ray diffraction is a useful tool to understand the internal structures of carbon fibers and graphitic lattice distance; crystalline size, and orientation of CFs have been estimated [6,7,8,9]. Typically, pitch-based CFs have a higher density with smaller lattice distance and larger crystalline structures than PAN-based CFs. High-temperature heat treatment to PAN-based CFs further increases the crystalline size with less lattice distance, hence higher mechanical properties are obtained [10]. Similar trends have also been observed in mesophase pitch-based CFs [11], which have confirmed the relationship between graphitic structure of CF and its resulting mechanical properties. Transmission electron microscopy (TEM) is a direct way to observe the graphitic structure of CF, and several groups have reported the internal structures of carbon fiber and interphase areas [12,13,14,15]. However, a detailed comparison between each precursor and its resulting carbon fiber TEM image and X-ray analysis is has not benn sufficient and still needs to be studied.

In this paper, we present high-resolution TEM images of PAN and a pitch-based CF prepared using a focused ion beam (FIB) and compare its wide-angle X-ray scattering results to gain an understanding of both graphitic structure and defect properties at the same time.

Furthermore, we accurately digitize the detailed microstructures into a finite element analysis (FEA) model. Modeling advancements have enabled accurate microstructures to be incorporated into simulations. Finite element squared (FE^2^) is a technique where microstructural finite element models are physically embedded into a larger finite element models defining the part geometry [16]. This method effectively replaces phenomenological constitutive models with discrete microscopic material models [17,18]. Multiscale modeling has been shown to be useful for the prediction of resultant composite performance. It has been investigated in applications scaled to a woven composite, chopped fiber, and porous composites [19,20,21,22,23]. In this work, the multiscale approach was used to explore smaller scales carbon fibers. Internal microstructural information was combined and digitized to generate a series of microstructures that accurately and statistically represented a given carbon fiber. Crystallite size, crystallite orientation, amorphous regions, and defects were all details that were measured using proven techniques. The representative digitized micro-structures were physically embedded into a larger single fiber model using FE^2^ and virtually tested under tension and compression. This digital twin approach was used for multiscale simulation of a single carbon fiber filament with controlled boundary conditions, in order to study the effect of the input graphitic crystal microstructure and dislocations between microstructures. In addition, the preliminary simulations were performed based on crystal size and the defects that correlated with the mechanical properties, such as skin/core misalignment. A ssociations between processing and the final material properties were elucidated and enabled the customization of microstructures for property targets. The method we used is defined as a digital twin approach because the experimental information describing the detailed graphitic crystal size, spacing, and alignment are represented by a local model of multiscale simulation. The geometric aspects of the internal carbon fiber microstructure have been well understood through X-ray and TEM and had not been digitized into a multiscale model as a sub-model characterizing single fiber mechanical properties before this work. The successful development of a digital twin model, including critical and fidelity microstructural features, could accelerate the fundamental understanding of comprehensive microstructure property relationship beyond tensile properties.

## 2. Materials and Methods

We selected two representative carbon fibers from PAN and pitch-based precursors. Scanning electron microscopy (SEM) was performed using a Helios G4 UC (Thermo Fisher Scientific, Waltham, MA, USA). This SEM is a multi-technique dual beam (electron and Ga ion) field-emission scanning electron microscope (FESEM) with a spatial resolution for imaging of 0.7 nanometers. Fiber tows were spread on a flat substrate and fixed with a diluted resin solution; then, thin lamellae were prepared using a FIB [24]. For the TEM observations, both cross-sectional and longitudinal directions of the CF samples were prepared and mounted on the TEM lift-out grid. The high-resolution transmission electron microscopy (HRTEM) image was obtained from a cold cathode field emission TEM, JEM-ARM200cF (JEOL), with 80 kV.

Fiber tows were mounted in the vacuum chamber, and wide-angle and small-angle X-ray scattering was performed using the Nanostar (Bruker, Billerica, MA, USA) with an Incoatec IµS microfocus X-ray source operating at 45 kV and 650 µA. The Cu Ka radiation beam (λ = 0.154 nm) was highly collimated with cross-coupled Göbel mirrors and a three pin-hole system. The wide-angle X-ray scattering (WAXS) pattern was captured by a film image plate and read with an FLA-7000 scanner (Fujifilm, Minato City, Tokyo, Japan).

Simulations were performed using MultiMechanics 19.0 TRUE Multiscale technology for FE^2^ execution, which were embedded representative volume elements (RVEs) modeling multiple scales simultaneously. The non-linear Newton-Raphson solver was used in the commercial software MultiMechanics (19.0, MultiMechanics-Siemens, Omaha, NE, USA). Tetrahedral elements were used for local and global scale models. The PAN-based carbon fiber model included 17,344 and 4200 elements for the global and local models, respectively. The pitch-based carbon fiber model included 1084 and 35,820 elements for the global and local models, respectively. Convergence tolerance for the solver was set at 5 × 10^−3^. Adequate mesh convergence was found, while mesh size was used explicitly as a digital twin aspect describing the crystal size differences. The rotation of the local scale crystal models was applied by separating the global scale into four skin/core rings and alternating positive and negative off-axis rotations. In the PAN-based model, off-axis rotations of +15% and −15% were applied to alternating skin/core rings. In the pitch-based model, the off-axis rotation of +1% and −1% was applied to alternating skin/core rings. This methodology was used for the skin/core alignment studies, as described in Section 3.4.1.

## 3. Results

### 3.1. SEM Results

**Figure 1:**
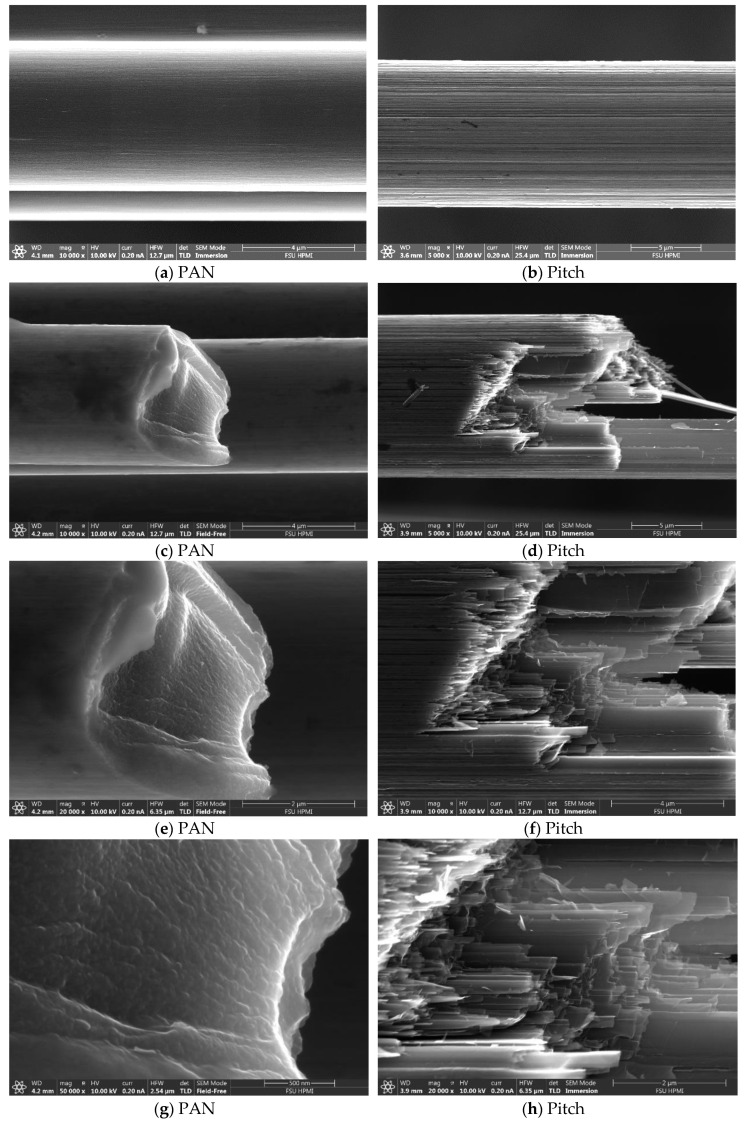
Scanning electron microscopy (SEM) of polyacrylonitrile (PAN) and pitch-based fibers in the (**a**,**b**) pristine forms and (**c**–**h**) fractured tensile forms.

The surface of the pristine PAN and pitch-based carbon fibers are shown in Figure 1a,b. The rougher texture of the pitch fiber is evident from the deeper ridges seen in the image contrast. The tensile fractured edge is visualized in Figure 1c–h. Through the fracture edge, a larger crystal can be seen in the pitch-based fiber as compared with the PAN-based fiber through the longer features and more angular fracture. In contrast, the PAN-based fiber has a smoother fracture due to the smaller crystal size formed through lower temperatures and a lower orientation than the pitch fibers [25,26].

### 3.2. TEM Results

**Figure 2:**
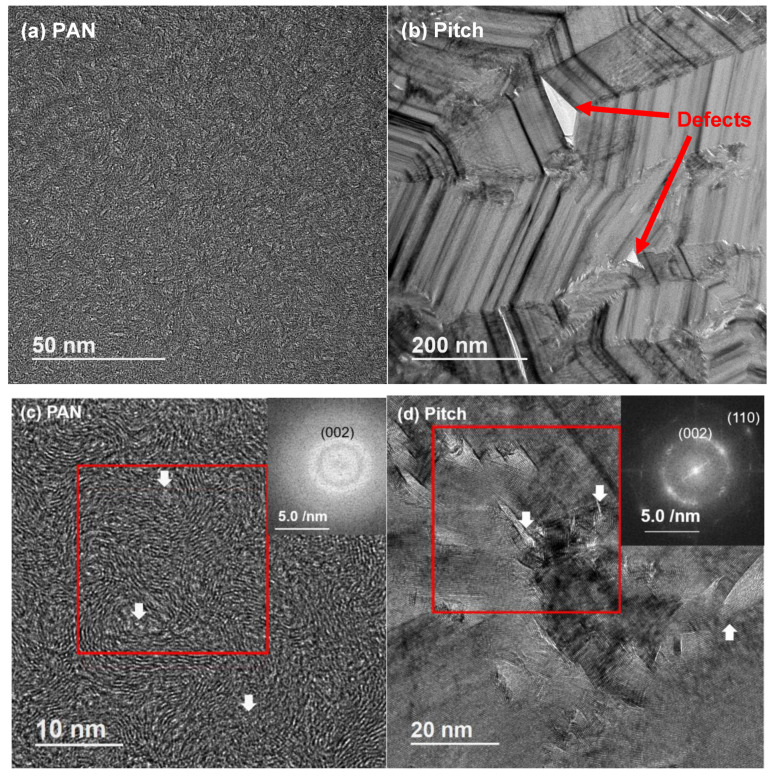
Cross-sectional TEM images of (**a**,**c**) PAN and (**b**,**d**) pitch-based carbon fiber at different magnifications. The inset shows FFT image of the squared area. The graphitic structure is more evident in the case of pitch fiber. Triangular defects between each crystalline structure can be seen in the pitch-based fiber.

HRTEM images of PAN and pitch-based CFs were taken in both the cross-sectional and longitudinal directions with respect to the fiber axis. Figure 2 shows the comparison of TEM images of each fiber’s cross-section at low and high magnification. Large crystalline graphitic structure from the pitch-based CF is clear from the low magnification image, and different sizes of triangular defects can be observed. High magnification TEM images show the size of the graphitic layer thickness to be around 2–3 nm for the PAN-based CF and 10–40 nm for the pitch-based CF, respectively. An inset in the high magnification TEM image show the fast Fourier-transformed (FFT) image of the selected square area in the TEM image. More random/amorphous orientation of graphitic structure in the PAN-based CF gives a circular pattern; meanwhile, the bright and large graphitic structure in the pitch-based CF gives peaks depending on the graphitic lattice directions.

**Figure 3:**
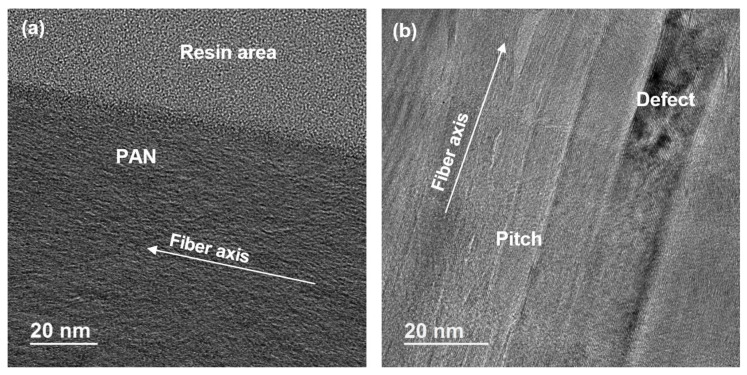
TEM images in the longitudinal direction of each fiber. (**a**) PAN-based CF; (**b**) Pitch-based CF with significantly longer and larger crystalline structures as compared with PAN.

Longitudinal direction TEM images along the CF axis are shown in Figure 3. Aligned graphitic planes can be easily identified for both fibers, and the pitch-based CF has clear graphitic structure. The pitch-based CF has more defects/void between crystalline structure, which can be observed in both Figure 2 and Figure 3.

### 3.3. X-ray Scattering and Orientation

**Figure 4:**
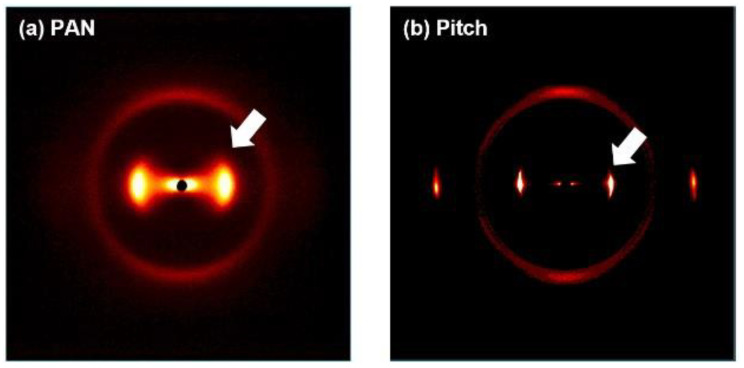
Wide-angle X-ray scattering (WAXS) for (**a**) PAN and (**b**) pitch-based carbon fiber. The white arrow is the location of the graphitic (002) peak around 26°.

X-ray scattering shows the crystalline structures and their characteristics. Fiber tows are located in the X-ray beam path, and its two-dimensional (2D) scattering patterns are collected using image plate and areal X-ray detector for wide-angle and small-angle scattering. Figure 4 shows the wide-angle X-ray scattering (WAXS) pattern, and from the center to radial direction, the 2θ and azimuthal (Φ) distribution can also be obtained from the intensity plot along the angle.

**Figure 5:**
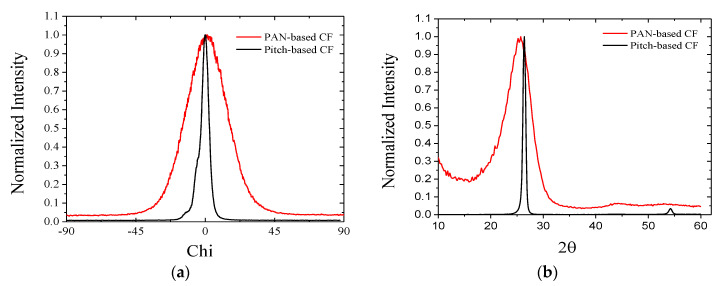
(**a**) Azimuthal angle distribution of intensity integrated from 20° < 2θ < 30° and (**b**) X-ray diffraction (2θ) data integrated from ± 5° (10 degrees) in the horizontal line (Φ = 0°).

The azimuthal intensity distribution, which is the integration of 20° < 2θ < 30°, is shown in Figure 5a. The pitch-based CF has a sharper and narrower distribution than the PAN-based CF in the azimuthal angle with a smaller residue. This means a higher alignment degree of graphitic planes along the fiber axis, which coincides with the TEM images with better alignment in the longitudinal direction, as shown in Figure 2 and Figure 3. The full-width half maximum (FWHM) in the azimuthal distribution is ~5.9° for the pitch-based CF and 31.6° for the PAN-based CF. The narrow distribution of the intensity means a higher alignment degree. The Herman’s orientation factor was derived from the FWHM and related fitting table [7], and the alignment factor was 0.99 for the pitch-based CF and 0.85 for the PAN-based CF.

Figure 5b is the X-ray diffraction along the horizontal line (Φ = 0°) with ± 5° integration, and similar trends with a narrow peak of pitch-based CFs can be observed. Graphitic (002) peak around 26° is marked with the white arrow in Figure 4. FWHM of (002) peak is 0.64° for the pitch-based CF and 6.07° for PAN-based CF, respectively. Similarly, the (100) peak, located at 42–45°, is also integrated along the vertical line (Φ = 90°) with ± 5°.

From the XRD, average interlayer spacing (d), crystal thickness (L_c_, (002) peak), and graphitic layer plane parallel to the axis (L_a_, (100) peak) are defined by the Bragg and Scherrer Equation [8]:

(1) $$d=\frac{\lambda }{2\sin\theta }$$

(2) $$L=\frac{K\lambda }{\beta \cos\theta }$$

where θ is the Bragg angle of peaks (°), λ is the wavelength of X-ray used (0.154 nm), and β is the FWHM (rad). The form factor K is 0.89 for L_c_, and 1.84 for L_a_. The resultant lattice spacing and crystal size are summarized in Table 1. The pitch-based CF was treated at a higher temperature, and a larger crystalline size was observed as a result [27].

The PAN-based CFs have a tensile modulus of 276 GPa and strength over 5.5 GPa [28,29]. Meanwhile, the pitch-based CF has a higher tensile modulus of around 965 GPa with a relatively low strength of roughly 3.1 GPa [30,31]. According to the internal structure from TEM and X-ray results, the higher modulus of pitch-based CF originates from the higher alignment of well-defined graphitic structure, and the increased strength of the PAN-based CF is the smaller crystalline size and the wavy pattern of misalignment between graphitic units. Therefore, achieving a highly aligned graphitic structure with an excellent pattern of misalignment is critical for high strength fiber, and adjusting the processing condition for these structure optimizations is vital for material design.

### 3.4. Digital Twin Model

**Figure 6:**
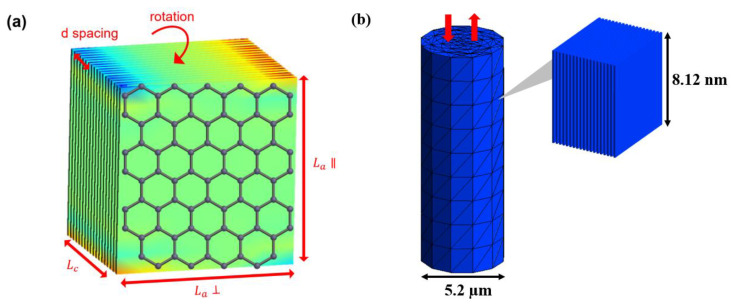
(**a**) Digitization of crystalline structure information; (**b**) Preliminary multiscale model for mechanical properties with RVEs, including crystalline structure.

Understanding the structure-property relationships of carbon fibers under tensile and compression is important for optimizing the microstructure through manufacturing processes, to improve carbon fiber and thus composite performance. FE^2^ was used along with the information found in Table 1 to create the local crystalline models inserted into the global single filament model. These representative digitized micro-structures are physically embedded into a larger single fiber model using FE^2^ for a single carbon fiber, as shown in Figure 6. The interactions between the crystal units are modeled as an interface between volume elements. The linear experimental results of the carbon fibers are potentially due to the inability to entirely control a single filament to the extent that progressive damage near the ultimate strength can be observed [32,33,34,35,36]. The benefit of the digital twin approach is that the loading conditions are perfectly controlled, and any potential nanoscale nonlinearity can be explored concerning the carbon fiber internal structure. The model can potentially provide a more precise study of the failure process.

By performing virtual testing using this method, the loading can be controlled and the desired properties obtained while circumventing many of the downfalls of experimental testing, mainly the instability of the unsupported fiber under compression [37,38]. Using virtual testing with a digital twin approach can be used for fundamental studies of significant factors that guide structure-property relationships which are not possible to do with a physical test. In this paper, the factors that are studied for PAN and pitch-based carbon fibers are alignment degrees, crystal size, skin/core alignment differences, and crystal unit to crystal unit dislocation strength.

**Figure 7:**
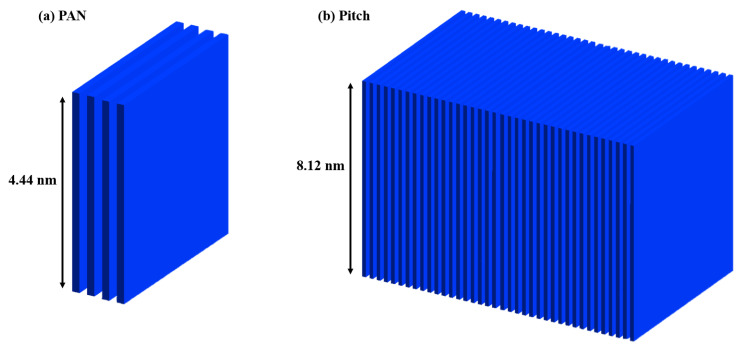
Digitization of crystalline structure information used as RVEs. (**a**) For PAN-based carbon fiber; (**b**) For pitch-based carbon fiber.

**Figure 8:**
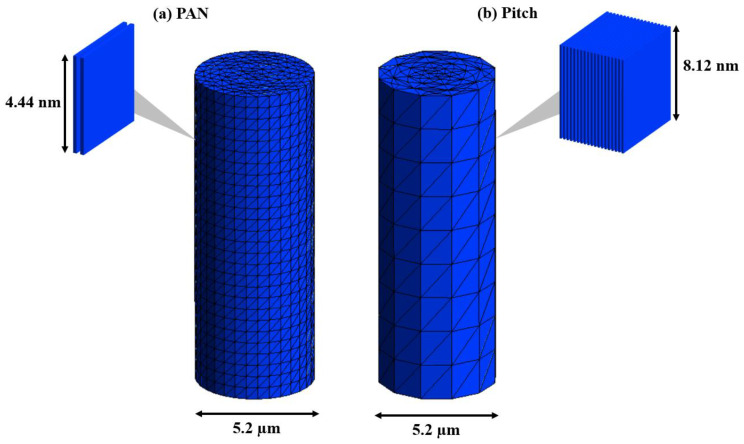
Global mesh sizing and multiscale RVEs from graphitic digitization. (**a**) PAN-based carbon fiber; (**b**) pitch-based carbon fiber.

The information from Table 1 was digitized into RVEs for PAN and pitch-based carbon fiber. The drastic size scale difference is visualized in Figure 7. The crystal unit for pitch is more than 16 times the size of the crystal unit for PAN when comparing Lc × La. This extreme size difference is seen in the SEM fracture in Figure 1c–h with the long sharp cracks in pitch-based and the shorter smooth breakage in the PAN-based carbon fibers. The unit size differences are digitized through mesh sizing in the single fiber global model seen in Figure 8.

Model factors were investigated for a significant impact on modulus and strength. While a reduction in orientation certainly reduces modulus, the aspect with the most direct effect on the strength and fracture mode is RVE to RVE dislocations, which simulate crystal unit to crystal unit cracks and sliding. These dislocations, or nano-cracks, influence the accumulation of nanoscale damage. From the X-ray experimental results shown in Table 1, it can be seen that the size of the RVEs and mesh sizes, noted in Figure 7 and Figure 8, controls the rate of the damage accumulation. A higher proportion of damage buildup directly results in earlier failure of the single fiber. The size of the PAN and pitch RVEs is seen in Figure 8. The crystal RVEs were halved for each model to reduce computation time.

**Figure 9:**
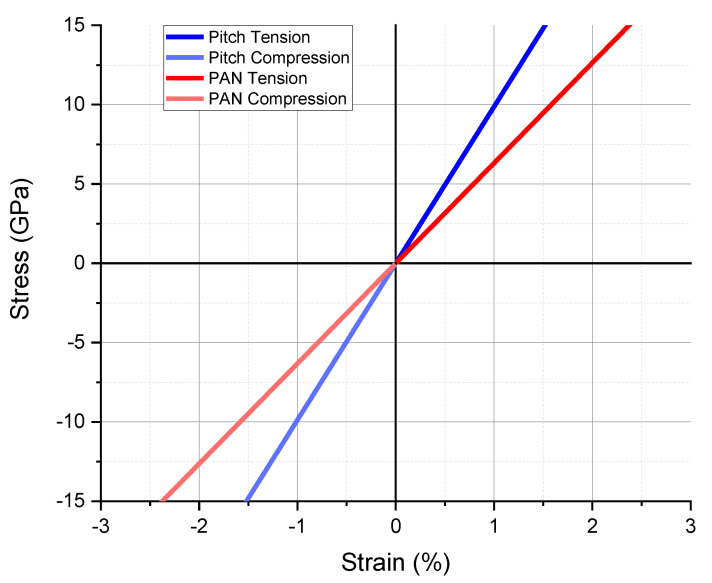
Tensile and compressive multiscale simulation results of baseline (no damage) properties for PAN and pitch-based single carbon fibers.

Tensile and compression multiscale simulations were performed for baseline properties without damage or failure criteria. The local scale crystal structure properties were assigned as linear elastic single-layer graphene with 1.02 TPa modulus [39,40,41]. The orientation factors of 0.85 and 0.99 for PAN and pitch, respectively, were applied to the rotation of the RVE. The results of tension and compression, assuming no damage or failure, have identical moduli and are shown in Figure 9 and Table 2. The baseline pitch-based carbon fiber simulation with no accumulating damage showed a tensile and compressive modulus of 987.5 GPa, which was slightly higher than reported values of 965 GPa [29,30,31]. The PAN-based carbon fiber simulation showed a modulus of 631.9 GPa, more than double that of the reported modulus of 276 GPa for such fibers [28,31]. For both PAN and pitch-based fiber models, the alignment degrees were modeled as alternating rings of negative and positive rotation throughout the cross-section of the fiber. Specifically, for PAN-based fibers, the 15% misalignment can only explain a 30–40% reduction in stiffness in simulation, whereas experimentally, there was a 50–60% decrease in modulus as compared with the pristine simulation case. The dislocation between crystal units and propagating fractures could be a reasonable explanation for this considerable reduction experimentally.

**Figure 10:**
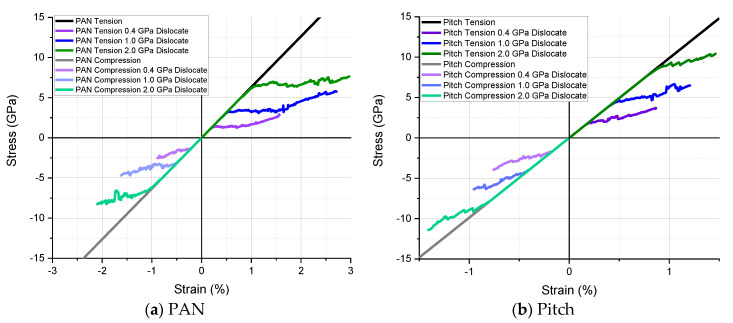
Tensile and compressive simulation stress/strain curves and final properties using crack dislocations between multiscale RVEs. (**a**) For pitch-based; (**b**) For PAN-based carbon fibers.

Due to the incredibly high strength of individual graphene sheets, reported at 130 GPa, lower stress conditions such as that experienced by carbon fiber cannot reach these environments to fracture the monolayer [39,40]. Other damage conditions must be considered in the model to capture the gap in measured and simulated strength accurately. RVE to RVE dislocations is an appropriate mode to study. This dislocation can be modeled as cracks between RVEs which results in progressive damage and stress reduction. This dislocation is modeled through crack initiation criteria in the microscopic case to study propagating fracture and the relation to single fiber tension and compression. The advantage is identifying progressive damage through an elastic crack simulation. The progressive damage near failure can occur too quickly at too low strains to study experimentally. The initiation criterion is given based on contact between two different objects (crystal units). Its methodology is based on defining interface elements from edges and surfaces expected to enter into contact during the simulation. Once the contact interface regions are defined, the elastic contact is assigned as the material model between crystal units. Elastic contact is used to model an interface between two bodies. This material type has to be associated with interface elements. Therefore, the meshes of the two bodies under consideration need to match each other in the region of contact approximately. The material interface is assigned to interfaces (edges or surfaces) between finite elements for automatic insertion of interface elements. The tangential and normal component of critical traction vectors in the r, n, and s-directions are the dislocation strengths. The dislocation strength between misaligned graphitic units was explored at 0.4, 1.0, and 2.0 GPa for the tangential component of critical traction vector in r, n, and s-directions (elastic contact strength) for investigation of progressive damage relationships to modulus and strength in tension and compression, as shown in Figure 10 and Table 3. The strength of the dislocation was found to have a significant influence on the final properties, and a dislocation value of 1.0 GPa in the PAN model resulted in a single fiber tensile strength of 5.8 GPa, which is consistent, and only slightly higher than literature results [28,41]. A dislocation value of 0.4 GPa in the pitch model resulted in a single fiber tensile strength of 3.8 GPa, which is also consistent and slightly higher than the literature [29,30,42]. An explanation for the predicted higher tensile strength in both PAN and pitch-based fibers is that it is due to not including voids or interface defects in the simulation. Allowing voids and interface defects, as seen in TEM results, likely reduces the strength and should be studied in future work. The higher dislocation value in the PAN fiber is likely caused by a small crystal unit wavy pattern of misalignment or unit cell bounding caused by a lower degree of alignment as compared with pitch fiber that can be seen through the SEM/TEM in Figure 1 and Figure 3. Tensile and compressive tests were simulated using these values, and the resultant performance is shown in Figure 10. The modulus is reported as the slope of the linear portion of the stress/strain graph.

**Figure 11:**
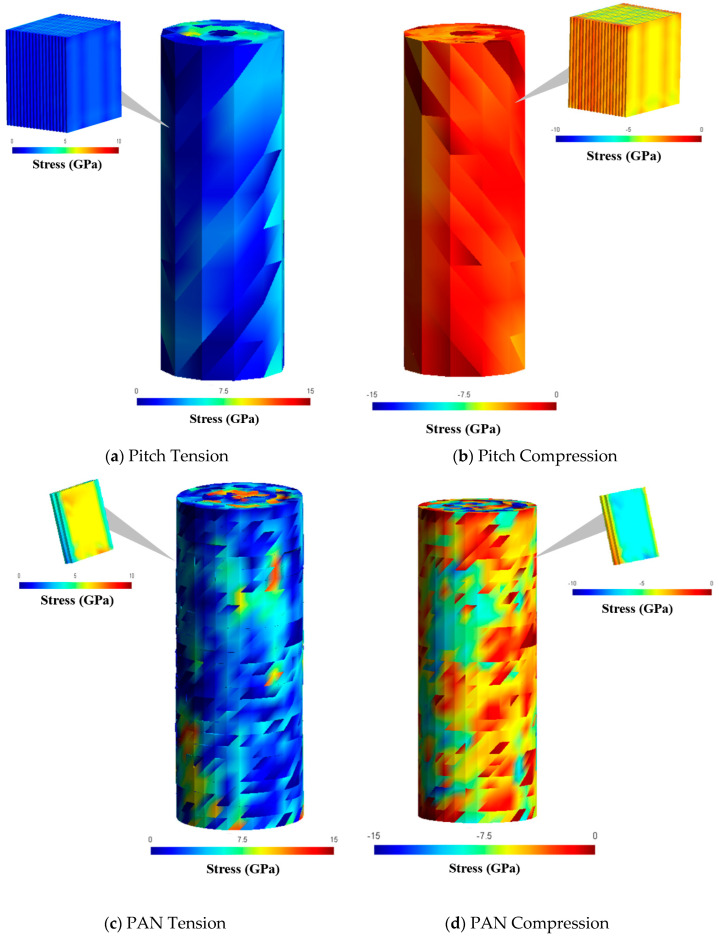
Simulation results for (**a**,**c**) tensile and (**b**,**d**) compressive deformation using crack dislocations between multiscale RVEs for (**a**,**b**) pitch-based carbon fibers and (**c**,**d**) PAN-based carbon fibers.

The local RVE scale was extracted from the global model stress/strain results shown in Figure 10. The multiscale simulations of tension and compression for pitch and PAN-based carbon fibers are seen in Figure 11 with a single local crystalline RVE pulled out for each model. Although only one RVE is visualized, the single filament model is represented as a multiscale model with the local graphitic RVE inserted at each element. The coarser pitch model has a lower strength due to the potentially lower dislocation saturation needed for the single filament to fully fracture as compared with the finer PAN mesh denoting smaller crystal unit size.

#### 3.4.1. Structure-Property Sensitivity

**Figure 12:**
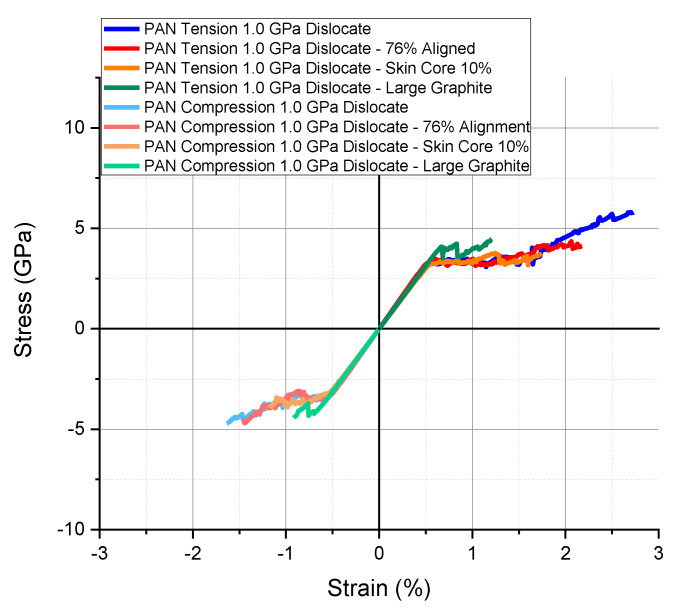
Tensile and compressive simulation results with microstructure modifications of alignment, skin-core alignment variations, and graphite size for PAN-based carbon fibers.

**Figure 13:**
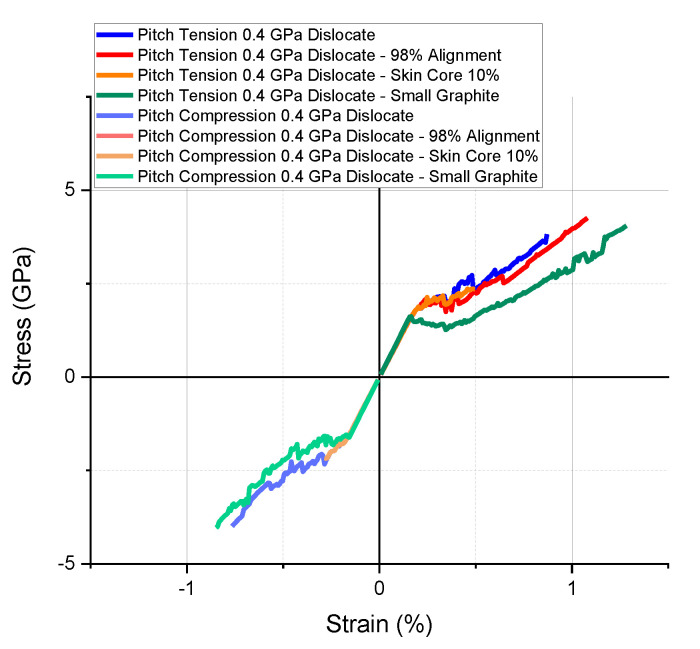
Tensile and compressive simulation results with microstructure modifications of alignment, skin-core alignment variations, and graphite size for pitch-based carbon fibers.

Additional factors that influence the modulus and strength of carbon fibers were explored. Figure 12 and Figure 13, Table 4 and Table 5 show the results for PAN and pitch-based fibers as compared with the dislocation strength between misaligned graphitic units that simulated the most accurate tensile strength. For PAN-based fibers, an additional 1% alignment results in a tensile and compressive strength reduction while increasing the tensile modulus. A skin/core alignment difference of 10%, with the more aligned graphite at the skin but still an average of 0.85% alignment as measured through X-ray, results in a tensile strength reduction of 36% and a compressive strength reduction of 17%. A more consistent alignment through the cross-section of the CF without sharp differences greatly influences the tensile strength. When the alignment degree increases by 1% in PAN-based carbon fibers, the modulus is predicted to increase by more than 4%. A skin/core misalignment of 10% shows a predicted decrease in tensile and compressive moduli by 3%. If the alignment degree, dislocation strength between misaligned graphitic units, and graphite properties of PAN-based fibers have a larger mesh size of pitch-based fibers, then the modulus, as well as tensile and compressive strength, are predicted to be slightly reduced due to increased slipping between 16 times larger crystal units represented by sized meshes.

In pitch-based fibers almost perfectly aligned at 99%, a reduction of 1% alignment results in an increase of 0.5 GPa in tensile strength and a 1% decline in tensile modulus as shown in Table 5. A skin/core alignment difference of 10%, with the more aligned graphite at the skin but still an average of 99% alignment as measured experimentally, resulted in a slight modulus decrease, a tensile strength reduction of 37%, and a compressive strength reduction of 45%. If the alignment degree, dislocation strength between misaligned graphitic units, and graphite properties of pitch-based fibers have a smaller mesh size of PAN-based fibers, then the modulus is predicted to be increased along with a tensile strength increase of 5% due to less drastic slipping between 16 times smaller crystal units represented by sized meshes. Manufacturing smaller crystal units could be used to target higher tensile strength markets, specifically as seen through SEM images in Figure 1.

## 4. Discussion

Representative CF materials from PAN and pitch-based precursors are selected to understand the microstructure-property relationships through a new digital twin approach. For this purpose, HRTEM images in both cross-section and longitudinal directions of the CF axis were taken. X-ray scattering was also performed to compare the crystalline size and alignment degree of the graphitic layer. Digital twin virtual multiscale modeling showed that PAN-based carbon fiber had a lower stiffness than pitch-based fiber, as expected. For PAN-based fibers, the dislocation, wavy pattern of misalignment, crystal unit sizes, interface imperfections, and potential voids between crystal units and propagating fractures are reasonable explanations for a reduction in stiffness and an increase in strength experimentally as comparing with that of pitch-based fibers. Since graphene properties can be assumed to be the same among the different types of carbon fibers, a plausible physics-based explanation for such a drastic decrease in modulus is dislocations between graphitic units. This is modeled in FE^2^ with the insertion of RVE to RVE cracks between elastic contacts. With the addition of the crack dislocation, the tensile strength of PAN and pitch-based fibers is reasonably estimated. For both the PAN and pitch-based carbon fibers, a 10% skin/core alignment difference negatively impacts the tensile strength by more than 15%. This bearing emphasizes the need for smooth alignment changes through the cross-section of the fiber to retain tensile strength benchmarks. Since carbon fiber properties are highly dependent on the internal structure, experimentally determining accurate microstructure and faithful digital representations are critical to ensure these virtual tests are predictive and informative to the manufacturing process by identifying critical structural factors that influence performance. Furthermore, by fine-tuning the digital twin model, we can gain new capability for simultaneously studying tension, compression, and shearing as well as thermal and electrical properties of carbon fiber in a single model.

## Figures and Tables

**Table 1:** Summary of crystalline structures and orientation from X-ray scattering.

Fiber Type	d-Spacing (nm)	L_c_ (nm)	L_a_ (nm)
PAN	0.352 ± 0.0005	1.48 ± 0.02	4.44 ± 0.16
Pitch	0.335 ± 0.002	12.97 ± 0.45	8.12 ± 0.96

**Table 2:** Summary of modulus results from Figure 9.

Property	Pitch		PAN	
	Tension	Compression	Tension	Compression
Modulus (GPa)	987.5	987.5	631.9	631.9

**Table 3:** Summary of modulus and strength results from Figure 10.

Model	Property	PAN		Pitch	
		Tension	Compression	Tension	Compression
Baseline	Modulus (GPa)	631.9	631.9	987.5	987.5
0.4 GPa Dislocation	Strength (GPa)	3.0	2.6	**3.8**	4.0
1.0 GPa Dislocation	Strength (GPa)	**5.8**	4.7	6.5	6.5
2.0 GPa Dislocation	Strength (GPa)	7.5	8.3	10.3	11.5

**Table 4:** Summary of modulus and strength results from Figure 12.

Model	Property	PAN	
		Tension	Compression
Baseline with Dislocation	Modulus (GPa)	631.9	631.9
	Strength (GPa)	5.8	4.7
+1% (86%) Alignment	Modulus (GPa)	658.8	658.8
	Strength (GPa)	4.1	4.6
Skin/Core 10% Misalignment	Modulus (GPa)	612.9	612.9
	Strength (GPa)	3.7	3.9
Pitch-Sized Large Graphite	Modulus (GPa)	625.9	625.9
	Strength (GPa)	4.5	4.4

**Table 5:** Summary of modulus and strength results from Figure 13.

Model	Property	Pitch	
		Tension	Compression
Baseline with Dislocation	Modulus (GPa)	987.5	987.5
	Strength (GPa)	3.8	4.0
−1% (98%) Alignment	Modulus (GPa)	981.4	981.4
	Strength (GPa)	4.3	1.9
Skin/Core 10% Misalignment	Modulus (GPa)	987.0	987.0
	Strength (GPa)	2.4	2.2
PAN-Sized Small Graphite	Modulus (GPa)	1025.9	1025.9
	Strength (GPa)	4.0	4.0
